# Photodynamic Inactivation Mediated by Endogenous Porphyrins of *Corynebacterium diphtheriae* in Planktonic and Biofilm Forms

**DOI:** 10.1021/acsomega.4c09308

**Published:** 2025-02-27

**Authors:** Gabriela Batista Alves, Mônica Regina da Costa Marques Calderari, Eduardo Nunes da Fonseca, Lincoln de Oliveira Sant’anna, Louisy Sanches dos Santos, Ana Luiza de Mattos-Guaraldi

**Affiliations:** † Laboratory of Diphtheria and Corynebacteria of Clinical Relevance, Rio de Janeiro State University, Avenue 28 de Setembro, 87—Fundos, 3° Andar, Vila Isabel, Rio de Janeiro CEP 20551-030, Brazil; ‡ Analytical Center Fernanda Coutinho, Rio de Janeiro State University, R. São Francisco Xavier, 524—Maracanã, Rio de Janeiro-RJ CEP 20550-013, Brazil; § General and Inorganic Chemistry Laboratory, Federal Institute of Education, Science and Technology of Rio de Janeiro, Rua Senador Furtado, 121, Maracanã, Rio de Janeiro, RJ CEP 20270-021, Brazil

## Abstract

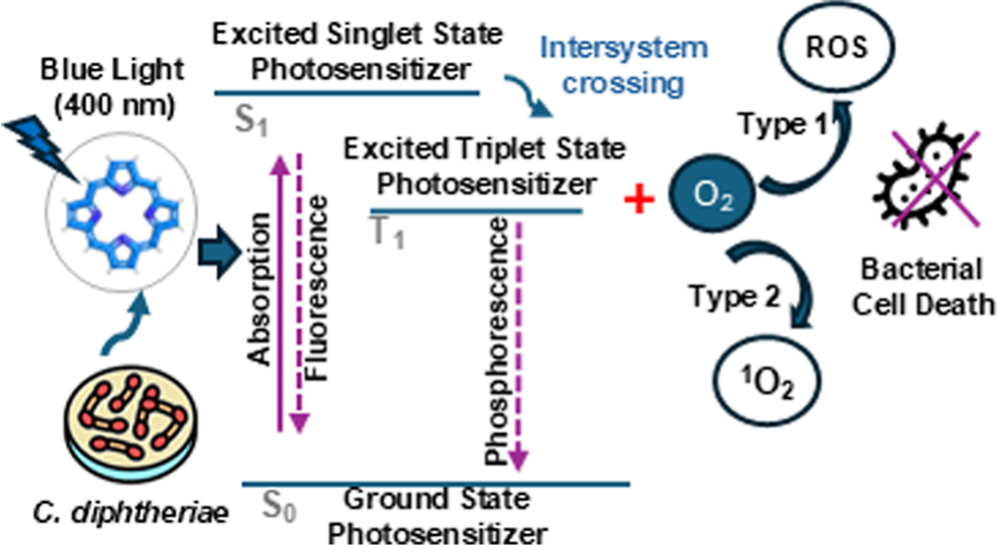

Photodynamic inactivation (PDI) has emerged as a promising approach to combat bacterial infections by using light activation of photosensitizers to induce microbial death. This study investigated the potential of endogenous porphyrins produced by *Corynebacterium diphtheriae* as photosensitizers for PDI. Qualitative analysis revealed the presence of porphyrins in all strains studied, with coproporphyrin III predominating. The addition of 5-aminolevulinic acid (ALA) enhanced porphyrin production, as evidenced by increased fluorescence intensity. In addition, high-performance liquid chromatography with diode array and mass spectrometry detection analyses confirmed the presence of coproporphyrin III and protoporphyrin IX in all strains, and the ALA supplementation did not alter the porphyrin profiles. Quantitative analysis showed that strain-dependent coproporphyrin III levels were significantly increased with ALA supplementation. Additionally, biofilm formation was positively correlated with porphyrin production, suggesting a role for porphyrins in biofilm formation. Photoinactivation experiments showed that the strains responded differently to light exposure, with ALA supplementation, reducing the time required for significant CFU/mL reduction. In addition, biofilm survival exceeded planktonic cell survival, highlighting the challenges posed by biofilm structures with regard to PDI efficacy. Despite the variable responses observed, all strains exhibited a reduction in viability following light exposure, demonstrating the potential of endogenous porphyrins for antimicrobial photoinactivation applications.

*Corynebacterium diphtheriae* is the causative agent of classic respiratory and cutaneous diphtheria, a toxemic disease whose prevention depends on the implementation of effective immunization programs by using diphtheria toxoid vaccines. In addition to diphtheria, systemic infections, often caused by nondiphtheria toxin producing strains, are increasingly observed. Cases of infections due to *C. diphtheriae* strains expressing resistance to antimicrobial agents used in therapies have also been reported.^1−4^ These facts demonstrated the relevance of additional research concerning innovative strategies for treating different types of infections caused by this pathogen, independent of diphtheria toxin production, and also to support antimicrobial therapies.

Photodynamic inactivation (PDI) of bacteria has been shown to be an efficient antimicrobial approach for killing antibiotic-sensitive bacteria as well as multidrug-resistant bacteria. The PDI technique uses the ability of photosensitizing molecules that, when exposed to visible light, moves to an excited state and transfers energy to molecular oxygen, generating reactive oxygen species (ROS) such as singlet oxygen, responsible for the oxidation of many biological molecules (enzymes, proteins, lipids, and nucleic acids) causing the death of the target pathogen.^5−7^ Two distinct types of reactions occur within the PDI process (Figure 1): type I reactions, which yield ROS such as superoxide (O_2_^–^) and hydroxyl radical (^•^OH), and type II reactions, which predominantly generate singlet oxygen (^1^O_2_). The products of these reactions can be produced simultaneously in PDI, with the proportion of each being dependent on the type of photosensitizer used and the ionic strength of its solvent.^8,9^

**Figure 1:**
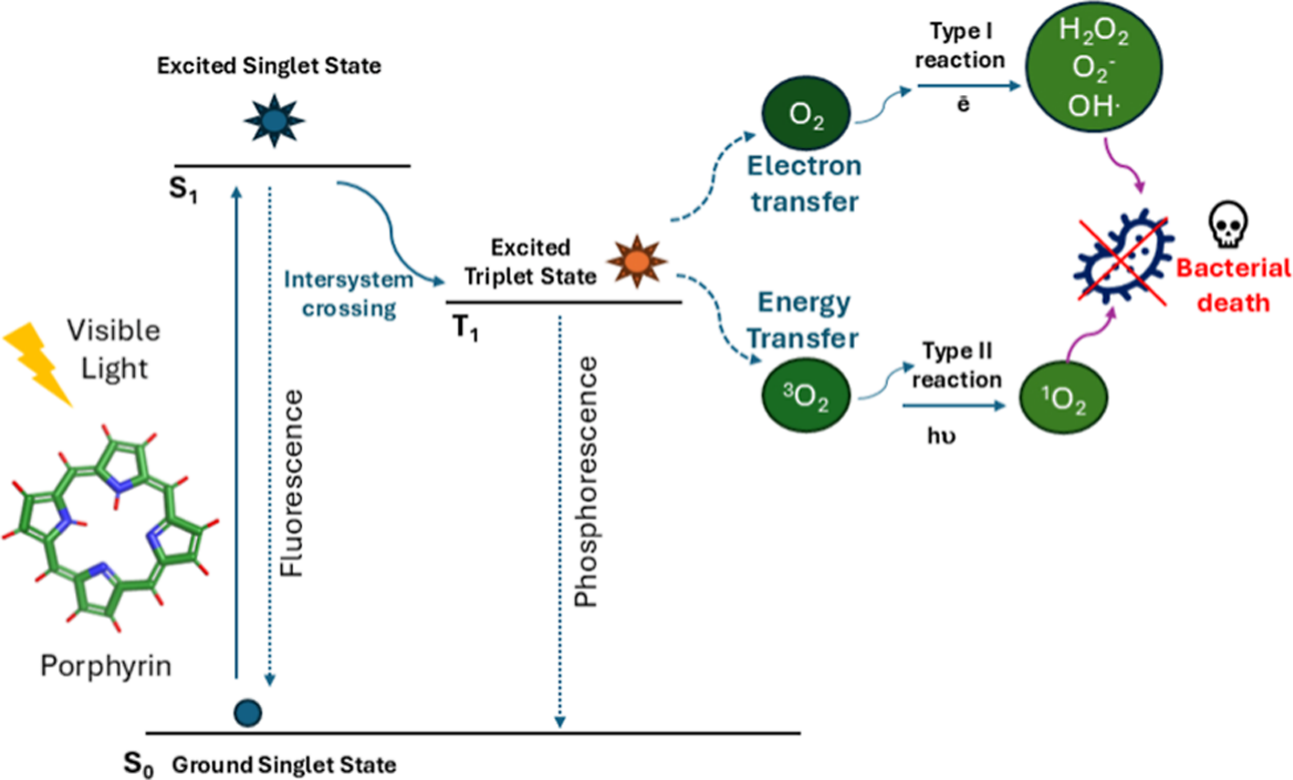
Jablonski diagram illustrates the mechanism of PDI, which can be categorized into two types of reactions. Type I reactions (electron transfer) occur when a photon is absorbed by the photosensitizer, in this case, porphyrins, leading to its excitation and transition from the ground singlet state (*S*_0_) to the excited singlet state (*S*_1_), and subsequently to the excited triplet state (*T*_1_) through intersystem crossing. In this excited state, porphyrins can transfer an electron (e^–^) to molecular oxygen (^3^O_2_), generating ROS such as superoxide anion (^•^O_2_^–^), hydroxyl radical (^•^OH), or hydrogen peroxide (H_2_O_2_). Type II reactions (energy transfer) follow a similar initial process. However, the photosensitizer in the triplet excited state (*T*_1_) transfers energy directly to molecular oxygen, resulting in the formation of highly reactive singlet oxygen (^1^O_2_).

Previous reported studies have shown that porphyrins are efficient photosensitizers due to their physical and chemical characteristics of absorbing light in blue part of the electromagnetic spectrum and are capable to transfer this energy to oxygen and generating ROS such as hydrogen peroxide and oxygen radicals.^10−13^ Their molecular structure allows them to efficiently absorb light in specific regions of the electromagnetic spectrum, featuring a primary absorption band known as the Soret band (located around 400 nm) and four smaller bands called Q-bands (ranging between 450 and 700 nm). The Soret band exhibits a high absorption intensity, which facilitates porphyrin excitation even with relatively low light doses, making it ideal for superficial applications. On the other hand, the Q-bands, although they have lower absorption intensity, allow the use of longer wavelengths that penetrate more effectively into deeper tissues. This combination of optical characteristics makes porphyrins versatile and effective photosensitizers in various clinical contexts, especially for the treatment of infections through PDI.^7,12,14^

Porphyrins are intermediate metabolites in the biosynthesis of vital molecules, such as hemeproteins. The heme group is essential to the function of these proteins, which are involved in energy generation by the electron transport chain, detoxification of host immune effectors, and other processes. The 5-aminolevulinic acid (ALA) is the early precursor of the heme group from bacterial and eukaryotic cells. The production of porphyrins is regulated by the ALA concentration. High levels of exogenously added ALA may lead to the accumulation and secretion of porphyrins that may be used as endogenous photosensitizers.^15−17^

For a long time, it was accepted that eukaryotes and prokaryotes used the same metabolic intermediates in their heme synthesis pathways, with protoporphyrin being the final intermediate in which the iron atom was inserted into protoporphyrin IX (PPIX) to produce protoheme (classical pathway). However, studies reported in recent years have shown that, in Actinomycetes and Bacillotes, the iron atom is inserted into coproporphyrin III, forming coproheme which is subsequently decarboxylated to form the protoheme group (Figure 2). This pathway, in which the iron atom is inserted into coproporphyrin III, is called the noncanonical pathway.^15,18^

**Figure 2:**
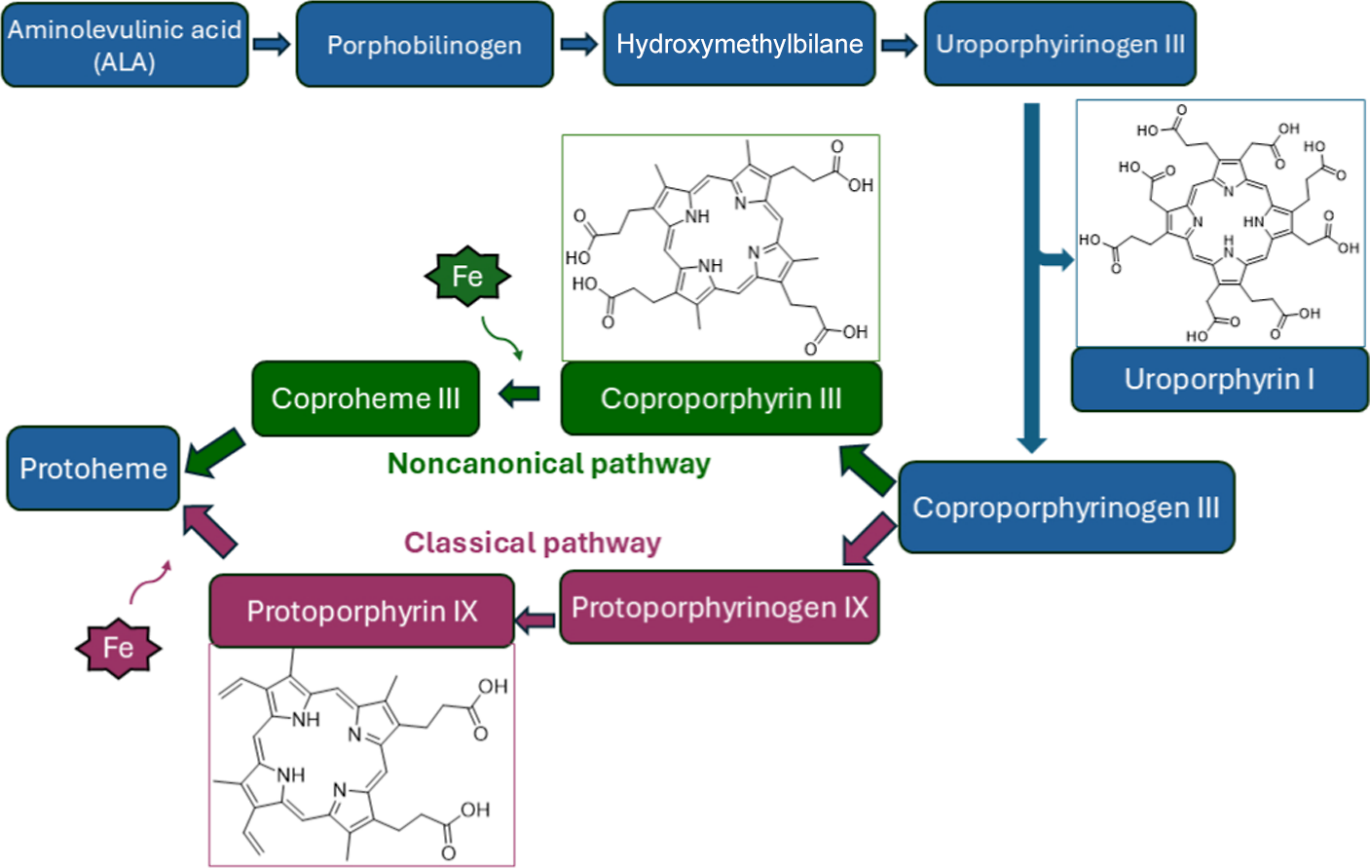
Bacterial heme biosynthesis. The heme synthesis pathway of most bacteria begins with the universal precursor ALA, and coproporphyrinogen III is formed through a series of conserved enzymatic steps. The classical pathway (magenta) forms heme through the PPIX intermediate; most organisms including Gram-negative bacteria and eukaryotes use this pathway. The noncanonical pathway (green), performed by most Gram-positive bacteria, produces heme through the coproporphyrin III intermediate. The chemical structures of uroporphyrin I (UPI), coproporphyrin III, and PPIX are also shown.

In the United States of America, during the year of 1930, the first reported research describing porphyrins produced by *C. diphtheriae* observed the presence of red and fluorescent pigments in filtered cultures.^19^ In the United Kingdom, during 1948, a reported study demonstrated the production of coproporphyrin III by *C. diphtheriae* strains.^20^ In Brazil, fluorescence assays by using Wood Lamp due to porphyrin production were reported as a novel approach for screening of *C. diphtheriae* strains during laboratorial diagnoses procedures in the 1980s.^21^ Chemical analysis of porphyrin extracts carried out in a recent study with strains of *C. diphtheriae* showed the predominance of coproporphyrin III in all tested strains. Furthermore, quantitative analysis revealed that ALA supplementation increased the amount of coproporphyrin III produced.^22^

Photoinactivation using the endogenous production of porphyrins used as photosensitizers molecules that have been shown to be effective for a variety of Gram-negative and Gram-positive bacteria.^23^ Studies have reported results for *Propionibacterium acnes* in the treatment of acne,^24,25^ in ulcers associated with *Helicobacter pylori*,^26−28^ and with *Salmonella enterica* strains.^29^ In rat models, PDI with ALA addition (ALA-PDI) has also been employed in vivo for the treatment of osteomyelitis with *Staphylococcus aureus* infections.^30,31^

There is a variety of responses to photodynamic therapy among the different species of bacterial pathogens, including total eradication and the absence of an effect from exposure to adequate light. Gram-negative bacteria are generally more resistant to PDI treatment. This can be explained by the difference in the structures of their outer membranes compared to those of Gram-positive bacteria. It is generally accepted that the peptidoglycan layer of Gram-positive strains has a much higher permeability than the outer membranes of Gram-negative bacteria, facilitating the absorption of ALA molecules and the flow of porphyrins produced by the investigated pathogen.^16,32^

The aim of this study was to verify the potential of coproporphyrin III produced by *C. diphtheriae* strains as a photosensitizer employing ALA and to correlate the degree of photodynamic response with the amount of coproporphyrin III produced by photoinactivation from bacterial planktonic cells and sessile forms from biofilms.

## Results and Discussion

### Qualitative Analysis of Porphyrin Production by *C. diphtheriae* Strains

The analysis of the porphyrin extracts from *C. diphtheriae* strains by HPLC-DAD confirmed the presence of coproporphyrin III in all three tested strains, and UPI was detected in only ATCC27010 and HC04 strains. However, PPIX was not detected in any of them. Retention times are shown in Table 1, and the chromatograms obtained on the HPLC-DAD are available in Figure S1 in the Supporting Information file.

**Table 1:** Retention Time of Porphyrin Standards and Extract of Porphyrins Obtained in HPLC-DAD^a^

standard/bacterial strains	uroporphyrin I (min)	coproporphyrin III (min)	protoporphyrin IX (min)
standards	5.75	9.09	11.7
ATCC27010	5.80	9.11	not detect
ATCC27010 with ALA	5.79	9.11	not detect
HC04	5.80	9.11	not detect
HC04 with ALA	5.79	9.11	not detect
2962	not detect	9.11	not detect
2962 with ALA	not detect	9.11	not detect

a *C. diphtheriae* strains were cultured in the absence and presence of ALA (2 mM).

The HPLC-MS analysis detected the *m*/*z* ratio for CPIII ([M + H]^+^ = 655) and the doubly charged parent ion ([M + 2H]^2+^ = 328.5) in total ion chromatogram (TIC) and specific ion monitoring for all strains studied. Enhanced product ion (^+^EPI) spectra obtained from the TIC of porphyrin extracts are available in Figures S2–S4 in the Supporting Information.

PPIX and UPI were not detected in the TIC of any strain. However, upon specific ion monitoring, the presence of their ions was detected in some strains, as shown in Table 2. Coproporphyrin III was identified in all strains and conditions, and the addition of ALA to the culture medium did not change the porphyrins profile, as evidenced by the chromatograms obtained of porphyrin extracts, available in Figures S5–S7, in the Supporting Information.

**Table 2:** Retention Time (min) of Porphyrin Standards and Extract of Porphyrins Obtained in HPLC-MS^a^

standard/bacterial strains	uroporphyrin I precursor ion (minutes) [M + H]^+^ = 830 (min)	coproporphyrin III precursor ion (minutes) [M + H]^+^ = 655 (min)	protoporphyrin IX precursor ion (minutes) [M + H]^+^ = 563 (min)
standard	10.83	15.74	28.47
ATCC27010	10.87	15.74	28.46
ATCC27010 with ALA	10.86	15.69	28.45
HC04	10.85	15.71	28.42
HC04 with ALA	10.86	15.70	28.42
2962	not detect	15.75	28.50
2962 with ALA	not detect	15.71	28.47

a *C. diphtheriae* strains were cultured in the absence and presence of ALA (2 mM).

The chromatograms of the extracts obtained from the ATCC27010 and HC04 strain cultures with the addition of ALA exhibited larger peak areas for the UPI, CPIII, and PPIX signals when compared to the extracts from the cultures without ALA, indicating an increase in the amount produced of the three porphyrins under investigation. The only exception was the PPIX peak area of sample HC04, which exhibited a decrease in the ALA culture as shown in Table 3.

**Table 3:** Peak Areas of Extract of Porphyrins Obtained in HPLC-MS^a^

standard/bacterial strains	uroporphyrin I peak area	coproporphyrin III peak area	protoporphyrin IX peak area
ATCC27010	2.05 × 10^6^	2.10 × 10^8^	2.50 × 10^7^
ATCC27010 with ALA	7.38 × 10^6^	4.64 × 10^8^	4.86 × 10^7^
HC04	3.57 × 10^6^	1.23 × 10^8^	4.02 × 10^7^
HC04 with ALA	1.22 × 10^7^	2.20 × 10^8^	1.54 × 10^7^
2962	not detect	6.01 × 10^7^	2.53 × 10^6^
2962 with ALA	not detect	1.14 × 10^8^	6.58 × 10^6^

a *C. diphtheriae* strains were cultured in the absence and presence of ALA (2 mM).

It has also been described that the type of porphyrins produced varies according to the duration of exposure to ALA. Short incubation times tend to accumulate more lipophilic porphyrins, such as PPIX, while longer periods result in a majority of hydrophilic porphyrins, such as coproporphyrin III and UPI.^9,33^ Future studies with different culture times and varying ALA concentrations are needed to detail the influence of this supplementation on the profile of the porphyrins produced by *C. diphtheriae*.

For a long time, it was believed that the heme group metabolic pathway was unique across the species. However, in the past decade, it has been shown that while the initial steps are conserved, the final stages can occur via two pathways. The classical pathway, found in some Gram-negative bacteria and eukaryotes, converts coproporphyrinogen III to PPIX, which then forms protoheme. The noncanonical pathway, present in Actinomycetes and Bacillotas, oxidizes coproporphyrinogen III to coproporphyrin III, then forms coproheme, which is decarboxylated to protoheme. Although the pathways have been identified, annotating the related genes remains challenging. Studies on *Bacillus subtilis* and *S. aureus* suggest that a single enzyme may act on both coproporphyrinogen and protoporphyrinogen, with a preference for coproporphyrin III.^15,17,34^

The detection of only coproporphyrin III in the TICs of all the strains studied shows the predominance of coproporphyrin III, reinforcing the hypothesis that the noncanonical heme group synthesis pathway is used. The analysis of specific ion monitoring was able to detect coproporphyrin III and PPIX in the extracts of all of the strains used in this study, corroborating the hypothesis of the dual function of coproporphyrinogen oxidase.

### Quantitative Analysis of Coproporphyrin III Produced by *C. diphtheriae*

Due to the predominance of coproporphyrin III in the extracts obtained from *C. diphtheriae* strains, calibration curves and validation of the method for its quantification by HPLC-DAD were performed. Statistical analysis was carried out by Action Stat software and using the linearity study (Ordinary Least Squares Method) using *p* < 0.05. The limit of detection (LOD) was determined in 0.27 μg and the limit of quantification (LOQ) in 0.82 μg. The standard deviation (SD) for calibration curve *A* was ±0.60329 μg/mL and for curve *B* was ±0.34159 μg/mL.

**Figure 3:**
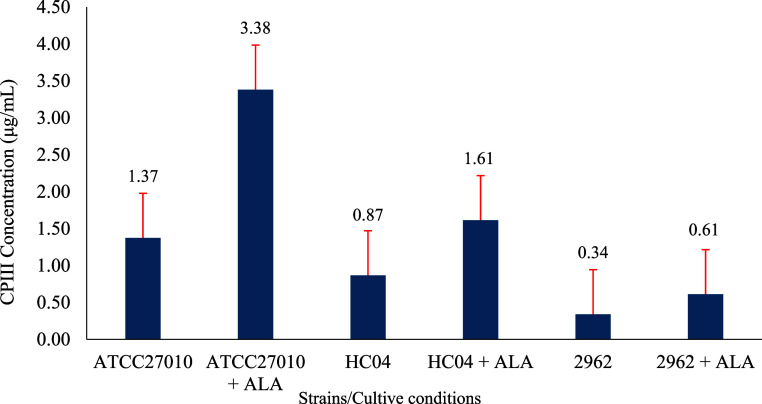
Quantification of coproporphyrin III produced by *C. diphtheriae* strains cultured in the absence and presence of ALA (2 mM). Quantification was performed by HPLC-DAD at the wavelength of 400 nm. SD = ± 0.60329 μg/mL. LOD = 0.27 μg/mL and LOQ = 0.82 μg/mL. The experiment was performed in triplicate for each strain and condition. CPIII, coproporphyrin III.

The quantification of coproporphyrin III in the studied extracts revealed significantly different values among *C. diphtheriae* strains used in the study, as shown in Figure 3. Supplementing the culture medium with ALA increased the amount of coproporphyrin III produced in all studied strains.

The influence of ALA supplementation on the profile of porphyrins produced by Gram-negative and Gram-positive bacteria has already been described in studies on the photoinactivation of microorganisms.^16^ For species such as *S. aureus*, *Staphylococcus epidermidis*, *Pseudomonas aeruginosa,* and *Escherichia coli*, it was observed that porphyrin synthesis peaked at concentrations of 1–2 mM ALA. However, the increase in the amount of porphyrin depends on the type of cell, with Gram-positive bacteria, being more likely to accumulate coproporphyrin III after ALA administration, while Gram-negative bacteria tend to accumulate other porphyrin intermediates.^16^

### Biofilm Formation

**Figure 4:**
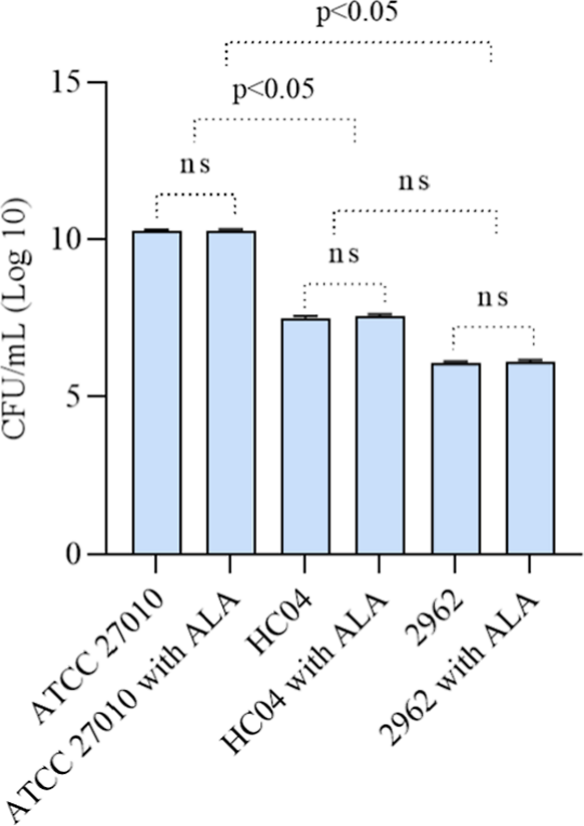
Biofilm cultured of *C. diphtheriae* strains cultivated in the absence and presence of ALA (2 mM). The addition of ALA to the cultures did not result in a notable change in the number of CFU/mL in the biofilms formed. The ATCC27010 strain produced more biofilm than the HC04 and 2962 strains. Statistical differences were evaluated through one-way analysis of variance (ANOVA), with a confidence level of 95% (*P* < 0.05).

The tests showed that all strains were able to form biofilm, and the addition of ALA to the culture medium did not change the amount of biofilm produced. Table 4 shows the average CFU/mL in the biofilm for each sample and study condition as well as the SD. Figure 4 shows the log 10 of the CFU/mL for each sample and cultivation condition, along with the results of the statistical tests, which is not statistically significant. The experiment was performed in triplicate for each strain and condition.

**Table 4:** *C. diphtheriae* Strains Cultured in the Absence and Presence of ALA (2 mM) in the Biofilm Formation Test^a^

strains/culture conditions	mean of CFU/mL	standard deviation (SD)
ATCC27010	1.80 × 10^10^	2.00 × 10^9^
ATCC27010 with ALA	1.90 × 10^10^	2.00 × 10^9^
HC04	3.23 × 10^7^	5.13 × 10^6^
HC04 with ALA	3.70 × 10^7^	6.08 × 10^6^
2962	1.20 × 10^6^	1.00 × 10^5^
2962 with ALA	1.27 × 10^6^	2.08 × 10^5^

a The experiment was performed in triplicate for each strain and condition.

The results point to greater biofilm formation by the strains that produce more coproporphyrin III in the cultures without the addition of ALA. However, the addition of ALA did not cause an increase in the biofilm formation by *C. diphtheriae* strains. Previous studies have shown that heme group metabolism is involved in signal transduction, acting as a cofactor for gas/redox sensors and modulating behaviors such as response to nitric oxide and biofilm formation.^35,36^ Data suggested that higher levels of biofilm formation by the strains that produce more coproporphyrin III may be linked more to the availability and presence of these signaling hemeproteins than to the amount of heme group precursors present in the culture medium.

### Photoinactivation of Planktonic Cells and Biofilm

**Figure 5:**
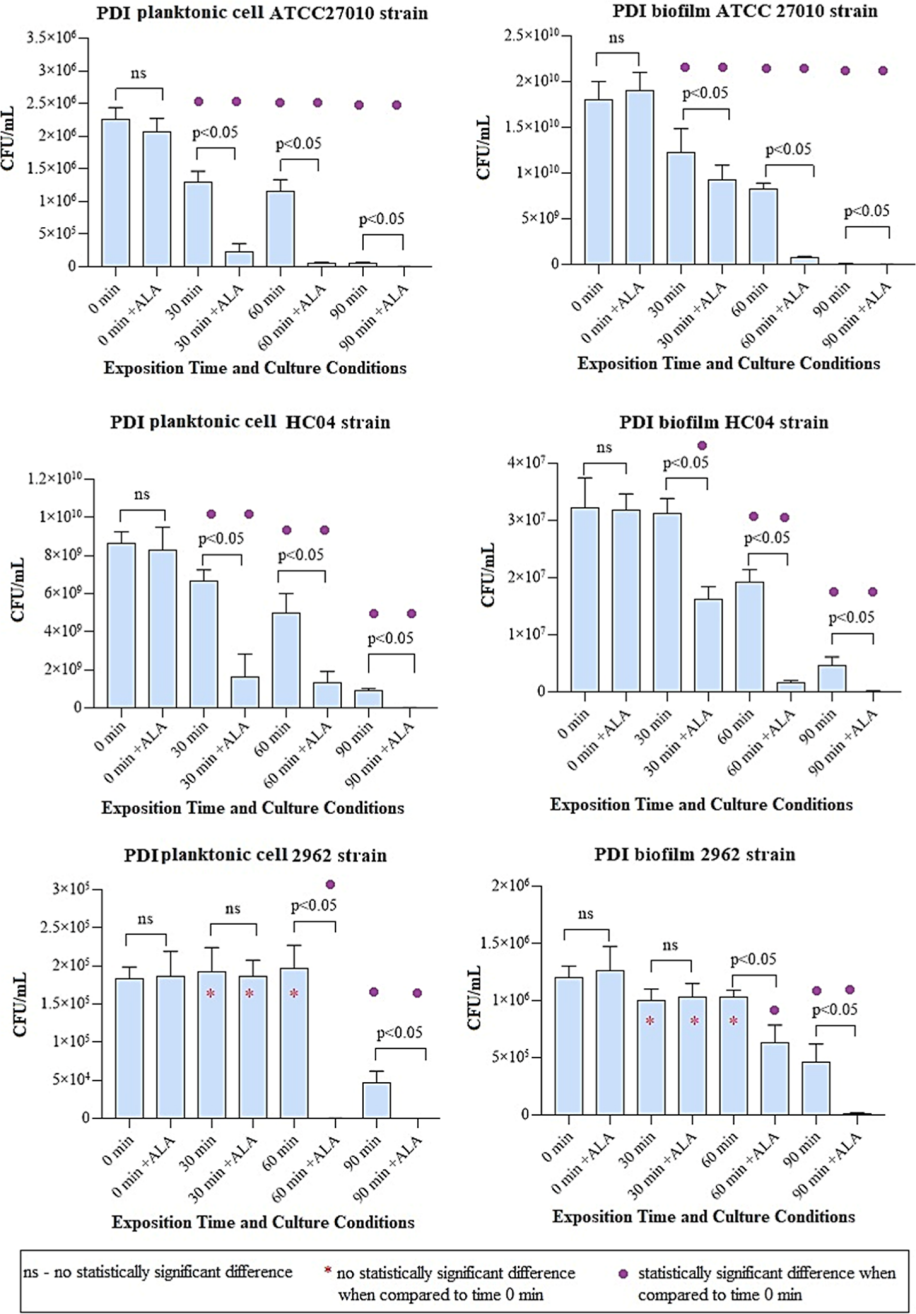
PDI of planktonic cells and biofilm of *C. diphtheriae* strains cultivated in the absence and presence of ALA 2 mM (^+^ALA) and using light exposure times of 30, 60, and 90 min. The results were compared with cultures not exposed to light (0 min). The results obtained in the cultures without and with ALA supplementation were also compared. Statistical differences were evaluated through one-way ANOVA, with a confidence level of 95% (*P* < 0.05). The experiment was performed in triplicate for each strain and condition.

All *C. diphtheriae* tested strains responded to some degree to exposure to light. Figure 5 shows the results of the photoinactivation of planktonic cells and biofilms. The time of exposure to light required to cause a reduction in the number of CFU/mL was directly correlated to the amount of CPIII produced by each strain. The ATCC27010 strain showed a reduction in the number of CFU/mL with only 30 min of exposure to light, in cultures without and with ALA. In contrast, 2962 strain, which produces much less CPIII, showed a reduction in the number of CFU/mL only after 60 min of exposure in the culture with the addition of ALA and 90 min of exposure in the culture without ALA.

The addition of ALA to the culture resulted in a reduction in the time of exposure to light needed to cause a significant decrease in the number of CFU/mL in both HC04 and 2962 strains. This reduction was observed in both experiments with planktonic cells and biofilms, with the time of exposure to light being reduced from 60 to 30 min for HC04 strain and from 90 to 60 min for the 2962 strain. In Table 5, the survival fraction in planktonic cells and biofilm cultures was presented, with and without the addition of ALA, after 90 min of exposure to light. The addition of ALA was observed to enhance the efficiency of photoinactivation for all of the strains and conditions studied. After 90 min of exposure, the cultures with added ALA had a reduction of 1–2 log of CFU/mL more than that of the cultures without ALA.

**Table 5:** Survival Fractions (*N*/*N*_0_) from *C. diphtheriae* Strains Cultures after 90 min of Light Exposure^a^^,^^b^^,^^c^

bacterial strain/type of cell	survival fraction (*N*/*N*_0_)	
	without ALA	with ALA
ATCC27010 planktonic cells	2.32 × 10^–2^	1.16 × 10^–3^
ATCC27010 sessile cells	6.85 × 10^–3^	1.23 × 10^–3^
HC04 planktonic cells	1.04 × 10^–1^	2.17 × 10^–3^
HC04 sessile cells	1.47 × 10^–1^	2.94 × 10^–3^
2962 planktonic cells	2.55 × 10^–1^	7.14 × 10^–4^
2962 sessile cells	3.89 × 10^–1^	1.05 × 10^–2^

a The strains were cultivated in the absence and presence of ALA (2 mM) and exposed to 400 nm wavelength light for 90 min.

b *N*_0_—CFU/mL before exposure to light dose.

c *N*—CFU/mL after 90′ of exposure to light dose.

A comparison of survival fraction between planktonic cells and biofilm revealed that except for strain ATCC27010, all exhibited an increase in biofilm survival. Previous studies have described that biofilm structures may affect the interaction of the photosensitizer as well as reduce the quantity of light reaching the bacteria, thereby decreasing the effectiveness of the photosensitizing process.^37,38^

This study showed a reduction in bacterial viability for all tested strains and culture conditions studied after exposure to LED light at 400 nm for both planktonic cells and biofilms. The strains studied showed different degrees of response to PDI. Several authors have previously described that the response to the PDI is markedly strain-dependent both in Gram-positive and Gram-negative bacteria.^39−42^ Therefore, although it was not possible to eradicate the strains at the exposure times and light doses studied, it is assumed that this can be overcome with adjustments to the light source, exposure time, and cultivation conditions.

The improvement of wavelengths used in photodynamic therapy is essential to maximize the treatment efficacy. A previous study that investigated the optimization of parameters for the photoinactivation of *S. aureus* infections on the skin demonstrated that the use of 400 nm light, which coincides with the Soret band of coproporphyrin III (CPIII), provides a significant reduction in bacterial viability in superficial infections due to its high absorption at this wavelength. The study also showed that wavelength multiplexing, combining blue light (400 nm) with green or red light (565 or 690 nm), increased the effectiveness of photoinactivation, resulting in additional reductions in bacterial viability. This synergistic approach increases the production of ROS, allowing infections at various depths to be treated more efficiently.^14^

Several factors have been identified as contributing to the susceptibility of this pathogen, including the composition of the cell wall, the levels and types of porphyrins, and the presence of protection mechanisms. A better understanding of the metabolism of the heme group and the mechanisms involved in the photoinactivation process is necessary and could lead to new methods to increase the effectiveness of antimicrobial PDI.

## Conclusions

In conclusion, the present study demonstrated the potential of porphyrins produced by *C. diphtheriae* strains as photosensitizers for photoinactivation, both in planktonic cells and biofilms. Moreover, the addition of ALA increased the production of coproporphyrin III, enhancing the efficacy of photodynamic therapy. Qualitative and quantitative analyses revealed strain-dependent variations on porphyrin production by *C. diphtheriae*, which correlated with the degree of bacterial susceptibility to photoinactivation. Data also suggested that biofilm formation may be influenced by the presence of porphyrins, indicating a potential role of these molecules in bacterial signaling pathways.

When planktonic cells and biofilms were compared, biofilm-associated cells exhibited greater resistance to photoinactivation, except for the ATCC27010 strain. This increased resistance is likely due to the protective structure of biofilms, which hinders light penetration and interaction with photosensitizers. Previous studies show that PDI can be an effective alternative to combat biofilm-associated infections, which are notoriously resistant to conventional antibiotics, and the optimization of the biofilm photoinactivation process is contingent upon a number of factors, including the composition of the biofilm and the penetration of light and photosensitizers.^43^

It has also been described that PDI can reduce the heterogeneity of bacterial resistance, making previously resistant subpopulations more susceptible to antibiotics, suggesting that photodynamic strategies can effectively complement traditional antimicrobial therapies and mitigate resistance diversity within bacterial populations.^44^

The findings underscore the necessity for further investigation into the composition of biofilms and their interaction with photosensitizing molecules. Additionally, there is a need to explore combined therapies, such as photodynamic therapy in conjunction with antibiotic therapy, to overcome biofilm resistance and enhance treatment efficacy.

This study has provided information about potential innovative strategies to combat *C. diphtheriae* and demonstrated a promising alternative to conventional antibiotics, particularly in the context of the growing threat of antibiotic-resistant pathogens. Future studies should focus on optimizing light exposure parameters and elucidating the mechanisms underlying bacterial photoinactivation. This will further advance the application of photodynamic therapy as a promising approach for controlling and treating bacterial infections, including those caused by *C. diphtheriae*.

## Methods

### Origin of Bacterial Strains

The origin and clinical and microbiological features of the *C. diphtheriae* strains used in this investigation are presented in Table 6.

**Table 6:** Origin, Clinical, and Microbiological Features of *C. diphtheriae* Strains Used in This Study^a^

strain	origin	*tox* gene
ATCC27010	type strain	negative
HC04	endocarditis (blood sample)	negative
2962	cutaneous diphtheria (skin)	negative

a ATCC, American Type Culture Collection (USA).

Experiments were performed with microorganisms from stock cultures grown in trypticase soy broth (TSB; Difco Laboratories, USA) at 37 °C for 48 h. Clinical samples were previously isolated from clinical specimens and identified as *C. diphtheriae* in the Laboratory of Diphtheria and Corynebacteria of Clinical Relevance, Faculty of Medical Sciences, Rio de Janeiro State University (LDCIC/UERJ), the Collaborating Center for Diphtheria Research/Ministry of Health, Brazil. Stock cultures in TSB medium supplemented with 20% glycerol were maintained at −70 °C. To confirm the identification of *C. diphtheriae* and to study DT production, multiplex polymerase chain reaction assays were performed as previously described with primer pairs targeting the following genes: *rpoB*, *dtxR*, and *tox*.^45^

### Culture Conditions and Porphyrin Extraction

The porphyrins produced in cultures without and with 2 mM ALA were analyzed. An inoculum of 3 mL was prepared for each strain in TSB with an optical density of 0.3 at 600 nm (OD_600_). Then, 1 mL was transferred to two flasks, each containing 50 mL of TSB. One was supplemented with 2 mM 5-aminulevulinic acid (ALA; Sigma-Aldrich, USA), and the other was not supplemented with ALA. The *C. diphtheriae* strains were added, and the flasks were incubated at 37 °C for 48 h. Then, bacterial growth was evaluated by measuring the OD_600_.

Porphyrins were then extracted from each bacterial suspension by using 25 mL of ethyl acetate/acetic acid (Sigma-Aldrich, USA) solution (1:4 v/v) and transferred to a 5 mL of 5% HCl (Sigma-Aldrich, USA), as previously described.^46^ The porphyrins extracted under the two culture conditions (without and with ALA supplementation) were analyzed using high-resolution chromatography coupled with UV absorption and mass spectrometry detectors.

### Preparation of Porphyrin Standard Solutions

The porphyrin standards used included UPI (Sigma-Aldrich, USA), coproporphyrin III (CPIII) (Santa Cruz Biotechnology, USA), and PPIX (Sigma-Aldrich, USA), which were dissolved in 5% HCl and remained stable for at least 24 h.

### Qualitative Analysis of Porphyrin Production

The three main intermediates in heme group biosynthesis (coproporphyrin III, UPI and PPIX) were analyzed in porphyrin extract from *C. diphtheriae* strains cultivated with and without ALA supplementation. The analytical techniques used were high-performance liquid chromatography (HPLC) coupled with UV absorption (HPLC-DAD) and mass spectrometry (HPLC-MS) detectors. All HPLC methods used during the present study were performed based on previously described assays^47^ with modifications detailed below.

HPLC-MS assays were performed by using PerkinElmer Altus Ultrahigh-performance liquid chromatography (UHPLC) systems (PerkinElmer, USA), including an autosampler, a column temperature controller, interfaced with an Altus SQ detector mass spectrometer, and an Altus UPLC BEH C18 column (5 μm particle size; 2.1 × 150 mm, PerkinElmer, USA) and connected to Empower 3.0 software (Waters Corporation, USA). The column temperature was 30 °C, and the porphyrins were separated by a 6 min gradient elution and a two-component mobile phase consisting of acetonitrile as solvent A and Milli-Q water acidified with formic acid 0.1% (Sigma-Aldrich, USA) as solvent B. Gradient elution commenced upon injection at 5:95 (A/B), which was changed to 100:0 (A/B) in 30 min and returned to initial conditions within 32 min. The column was allowed to equilibrate for 5 min at 5:95 (A/B) before the next injection, and the flow rate used was 0.3 mL/min, with a maximum flow rate of 0.5 mL/min. The capillary voltage was 3.5 kV, with a cone voltage per tune, a desolvation temperature of 450 °C, a source temperature of 150 °C, a carrier gas flow (nitrogen) of 800 L/h, and in the cone rate of 40 L/h.

Continuum data were acquired in the positive-ion mode, the ratios of mass (*m*/*z*) of 563 (PPIX), 655 (CPIII), and 830 (UPI) were monitored, and the TICs were verified.

HPLC-DAD experiments were carried out with an Agilent 1200 HPLC instrument (Agilent Technologies, USA), including an autosampler, a column temperature controller, and a diode array detector, connected to Agilent ChemStation software. Porphyrins were separated on a Hypersil Gold C18 column (5 μm particle size; 250 mm × 4.6 mm, Thermo Fisher Scientific Inc., USA). The mobile phase was composed of methanol as solvent A and ammonium acetate as solvent B. Gradient elution commenced upon injection of 20:80 (*A*/*B*), which was changed to 50:50 (*A*/*B*) in 3 min and 100:0 (*A*/*B*) in 7 min, and between 10 and 17 min, the initial conditions of 20:80 (*A*/*B*) were maintained. The column was allowed to equilibrate for 3 min with 20:80 (*A*/*B*), before the next injection, and the flow rate used was 0.7 mL/min. The column temperature was 30 °C. The excitation and emission wavelengths used in the fluorescence detector were 395 and 630 nm, respectively, and the PMT gain factor of 13. With a multichannel UV detector, the wavelength of λ = 400 nm was monitored and UV spectra between 190 and 640 nm were recorded.

### Quantitative Analysis of Coproporphyrin III

Coproporphyrin III was quantified by HPLC-DAD, employing the identical apparatus and methodology previously delineated in this study for the qualitative analysis of porphyrins. Initially two different calibration curves were prepared from coproporphyrin III maintained in a stock solution of 1000 μg/mL in 5% HCl: curve *A*—one from 2 to 10 μg/mL; curve *B*—a second from 10 to 50 μg/mL. Both curves were performed in triplicate. Chromatographic peak area abundances (based on HPLC-DAD chromatograms) were obtained using ChemStation software (version 10.1, Agilent Technologies), where the peaks were integrated. The peak area table was exported to Microsoft Excel for quantitation. Statistical analysis was carried out by Action Stat software and using the linearity study (Ordinary Least Squares Method) using *p* < 0.05 and the LOD, and Limit of Quantitation (LOQ) were calculated.

### Photoinactivation of Planktonic Cultures

The photoinactivation tests on planktonic cultures were carried out without and with ALA supplementation (2 mM) and using light exposure times of 0 (no light exposition), 30, 60, and 90 min. The effect of ALA supplementation on the culture medium was also analyzed by comparing the results between cultures without and with the addition of ALA.

The methodology used was carried out based on previously described method.^33^ An inoculum of 3 mL was prepared for each strain in TSB with an optical density of 0.3 at 600 nm (OD_600_). Then, 1 mL of this inoculum was transferred to two separate flasks, each containing 50 mL of TSB. One flask was supplemented with 2 mM ALA, while the other remained unsupplemented. The *C. diphtheriae* strains were cultured in these flasks and incubated at 37 °C for 48 h. After the incubation period, the bacterial growth was evaluated by measuring OD_600_.

Subsequently, 1000 μL of the 48 h cultures (from the 50 mL flasks) was transferred into 24-well plates. For each strain, two plates were prepared: one containing the culture supplemented with ALA and the other without ALA supplementation.

The 24-multiwell containing bacteria were illuminated from above at a distance of 10 cm during 30, 60, and 90 min. The temperature was monitored and kept at 20 °C. The light array consisted of two 400 nm/7 W LED lamps with a fluence rate of 155 mW/cm^2^. The number of CFU/mL was determined after plating an aliquot of serial dilutions on agar plates.

All experiments were carried out in triplicate. ANOVA and Student’s *t*-test were used to determine statistical significance employing GraphPad Prism 9 software. A value of *p* < 0.05 was considered significant.

### Biofilm Formation and Photodynamic Inactivation

The photoinactivation tests on biofilm cultures were carried out without and with ALA supplementation (2 mM) and using light exposure times of 0 (no light exposition), 30, 60, and 90 min. The effect of ALA supplementation to the culture medium was also analyzed by comparing the results between cultures without and with the addition of ALA.

The methodology used was carried out according to the previous study with the changes described below.^33,48^ An amount of 1000 μL of bacteria (OD_600_ of 0.3) in TSB was placed into each well of 24-multiwell plates, containing a plastic Thermanox coverslip in each well. Two plates were prepared for each strain, one supplemented with 2 mM ALA and the other without ALA supplementation. After 48 h of incubation, the 24-multiwell containing bacteria were illuminated from above at a distance of 10 cm during 30, 60, and 90 min. The temperature was monitored and kept at 20 °C. The light array consisted of two 400 nm/7 W LED lamps with a fluence rate of 155 mW/cm^2^.

The number of CFU/mL was determined after plating an aliquot of serial dilutions on agar plates. Then, each individual Themanox coverslip was transferred to a sterile plastic tube of 15 mL containing 5 mL of phosphate-buffered saline at pH 7.2 and immersed in an ultrasonic bath (E15-H; Elmasonic, Singen, Germany). Sonication was carried out at 37 kHz with an output power of 240 W, at 37 °C for 5 min. After sonication, the coverslips were discarded, and the number of CFU/mL was determined after plating an aliquot of serial dilutions on the agar plates.

All experiments were carried out in triplicate. ANOVA and Student’s *t*-test were used to determine statistical significance employing Graphpad Prism 9 software. A value of *p* < 0.05 was considered significant.

## Supporting Information Available

Chromatograms and spectra from HPLC-MS and HPLC-DAD analyses (PDF)
