# Supplementary material for: Photodynamic Inactivation Mediated by Endogenous Porphyrins of Corynebacterium diphtheriae in Planktonic and Biofilm Forms
Source: ACS Omega. 2025 Feb 27;10(9):9177–86. doi: 10.1021/acsomega.4c09308 (PMC11904688; doi:10.1021/acsomega.4c09308)
Supplement: Supplementary file 1 — ao4c09308_si_001.pdf [file ao4c09308_si_001.pdf]

Supporting Information

Photodynamic inactivation mediated by endogenous porphyrins of *Corynebacterium diphtheriae* in planktonic and biofilm forms

Gabriela Batista Alves[1,3,*], Mônica Regina da Costa Marques Calderari[2], Eduardo Nunes da Fonseca[2], Lincoln de Oliveira Sant'anna[1] Louisy Sanches dos Santos[1], Ana Luiza de Mattos-Guaraldi[1]

[1] Laboratory of Diphtheria and Corynebacteria of Clinical Relevance, Rio de Janeiro State University, Av. 28 de Setembro, 87 - Fundos, 3° andar. Vila Isabel, RJ, Brazil, CEP 20 551-030

[2]Analytical Center Fernanda Coutinho, Rio de Janeiro State University, R. São Francisco Xavier, 524 - Maracanã, Rio de Janeiro - RJ, Brazil, CEP 20550-013

[3] General and Inorganic Chemistry Laboratory, Federal Institute of Education, Science and Technology of Rio de Janeiro, Rua Senador Furtado, 121, Maracanã, Rio de Janeiro, RJ, Brazil, CEP 20270-021

Corresponding Author E-mail: gabriela.alves@ifrj.edu.br

Contents

Supplementary Figures - Chromatograms obtained from HPLC-DAD and HPLC-MS analyses and Enhanced Product Ion (+EPI) spectrum obtained from HPLC-MS of porphyrin extracts from *C. diphtheriae* strains grown without and with the addition of ALA (2 mmol/L).

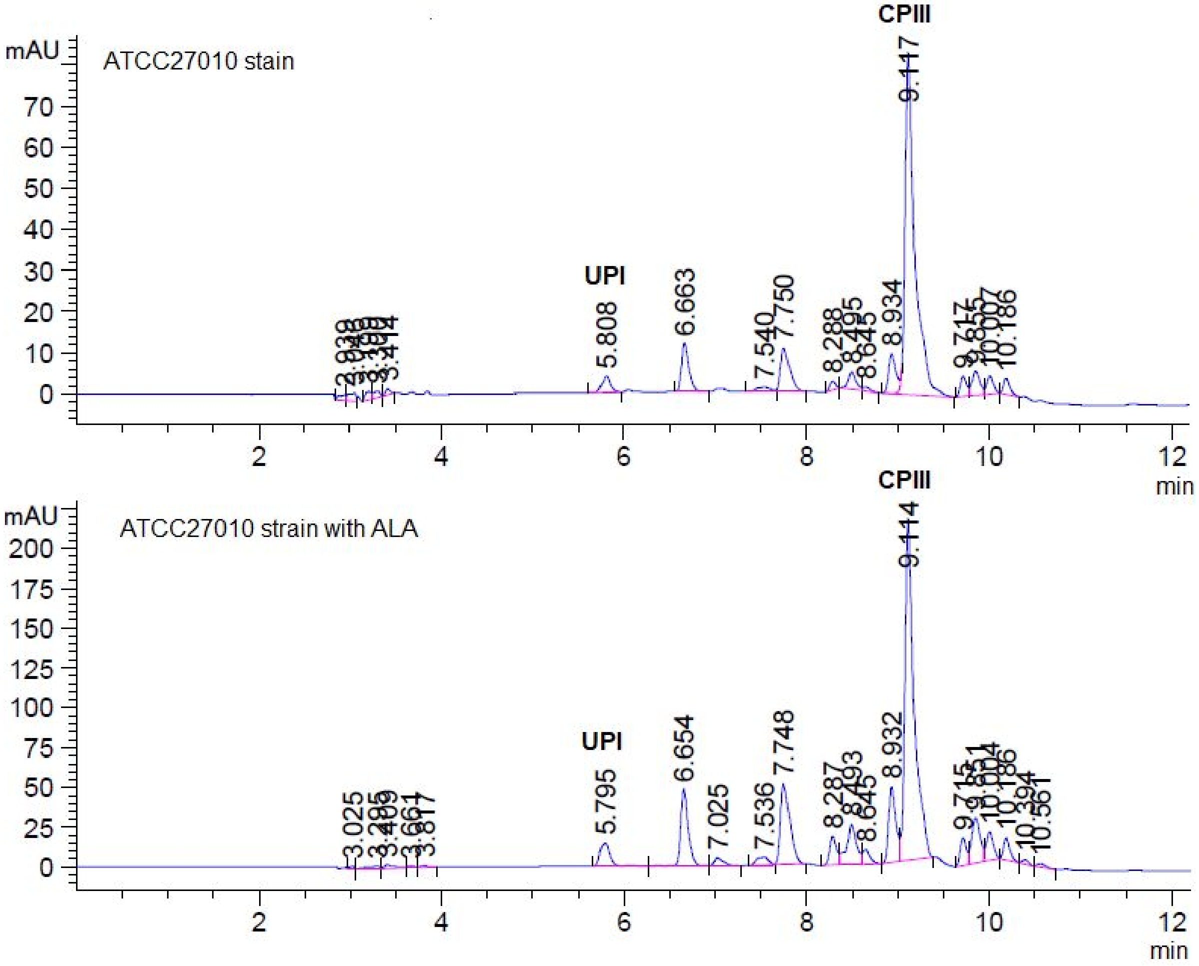

Figure S1. Chromatograms obtained in HPLC-DAD from porphyrin extracts from *C. diphtheriae* ATCC27010 strain cultivated with and without ALA (2 mmol/L) addition.

CPIII, copropophyrin III; UPI, uroporphyrin I; PPIX, protoporphyrin IX.

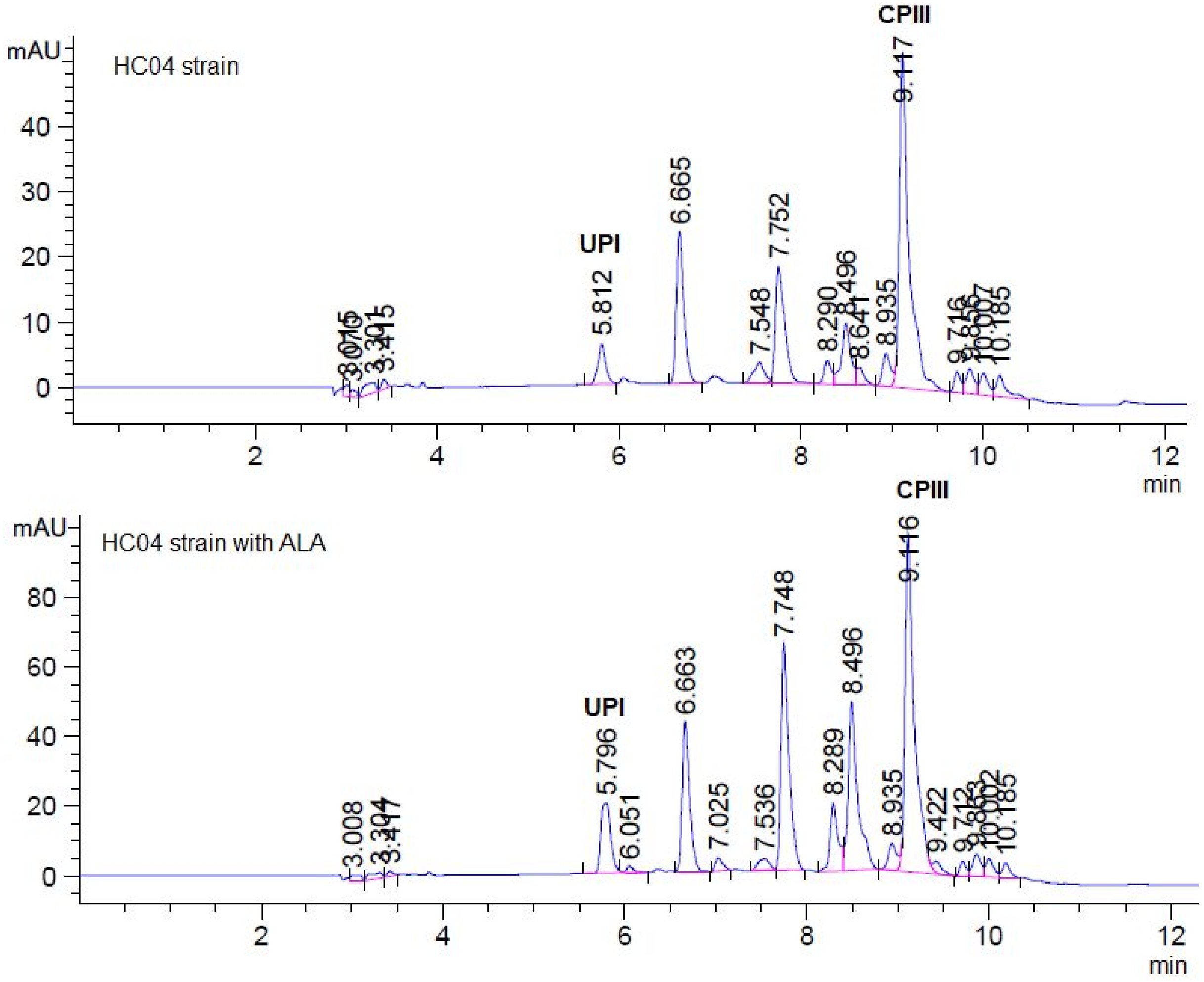

Figure S2. Chromatograms obtained in HPLC-DAD from porphyrin extracts from *C. diphtheriae* HC04 strain cultivated with and without ALA (2 mmol/L) addition.

CPIII, copropophyrin III; UPI, uroporphyrin I; PPIX, protoporphyrin IX.

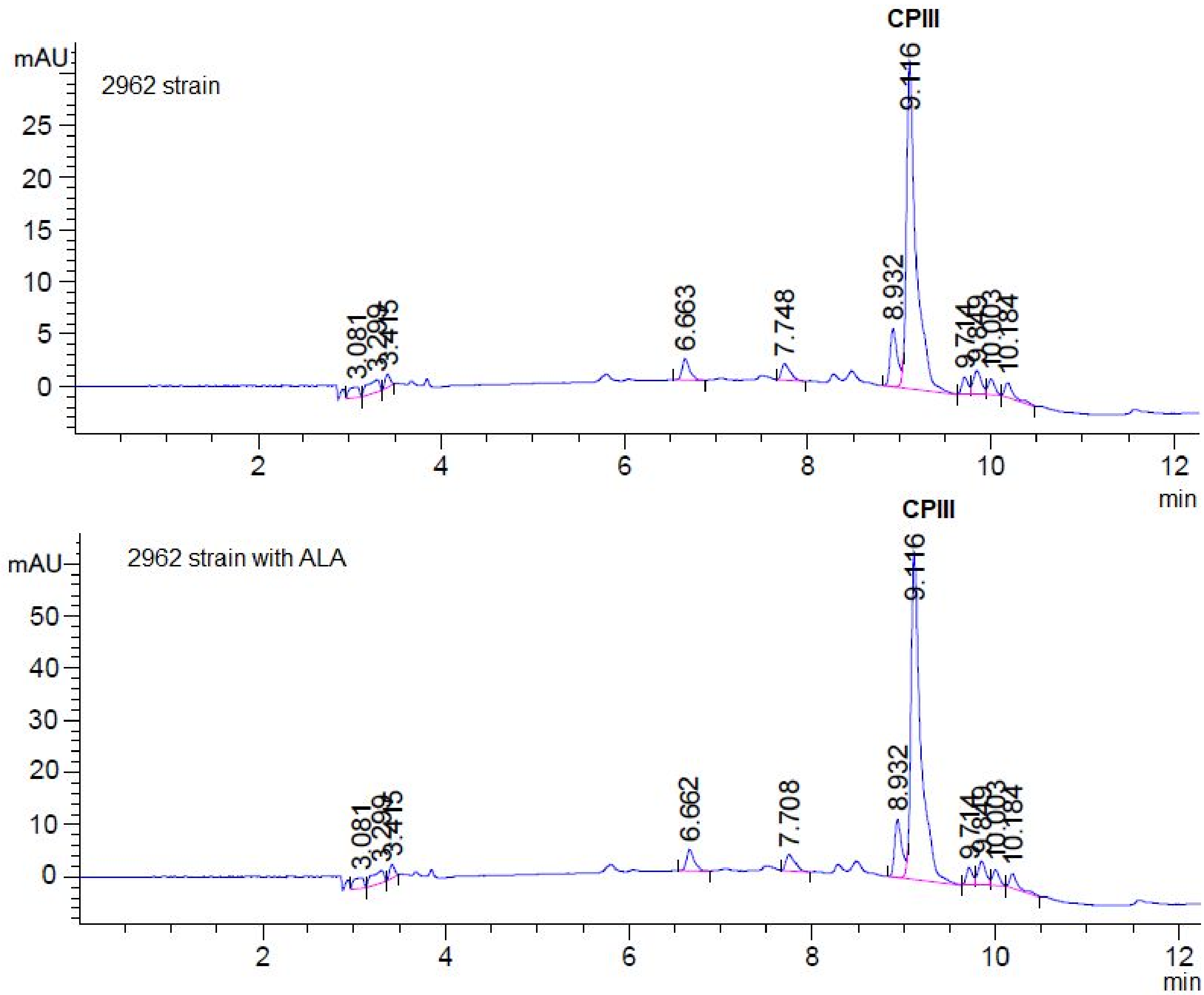

Figure S3. Chromatograms obtained in HPLC-DAD from porphyrin extracts from *C. diphtheriae* 2962 strain cultivated with and without ALA (2 mmol/L) addition.

CPIII, copropophyrin III; UPI, uroporphyrin I; PPIX, protoporphyrin IX.

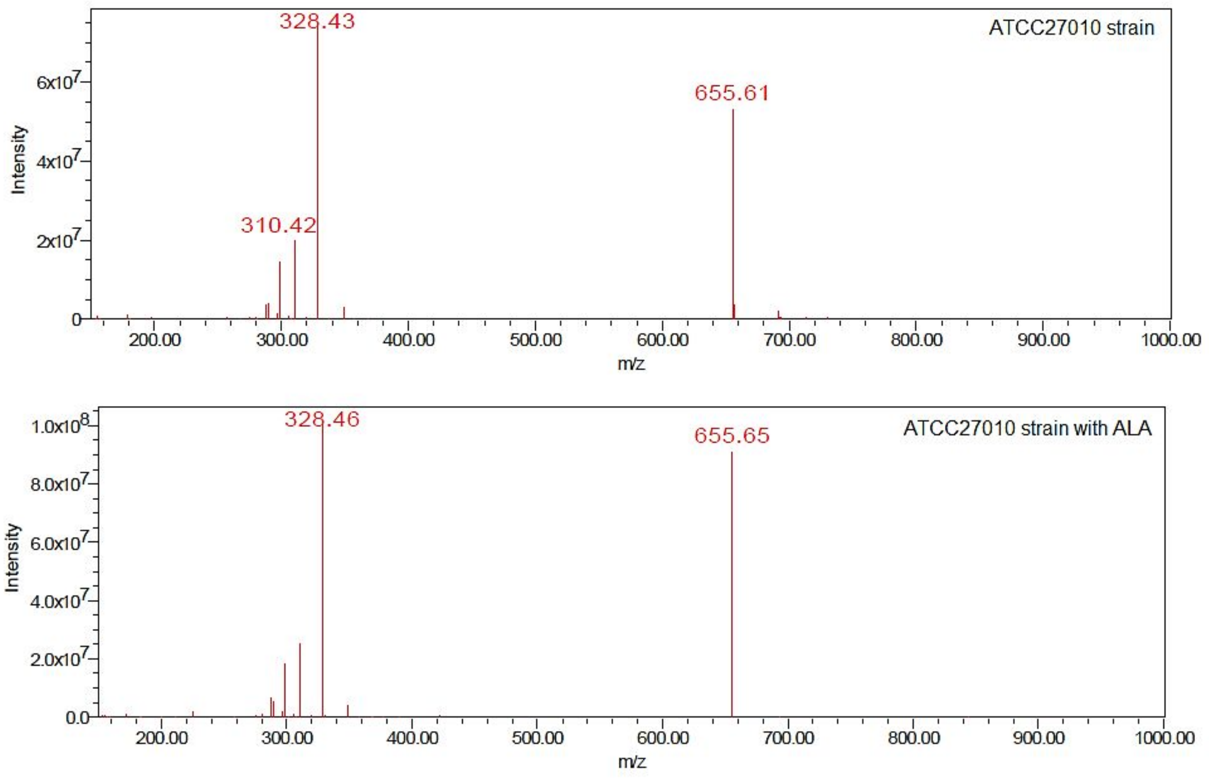

Figure S4. Enhanced product ion (+EPI) spectrum taken from TIC of porphyrin extracts from ATCC27010 *C. diphtheriae* strain cultivated with and without ALA (2 mmol/L) addition. The spectrum shows the m/z precursor ion ($[M+H]^+$ = 655) and the doubly charged parent ion ($[M+2H]^{2+}$ = 328.5), both for coproporphyrin III.

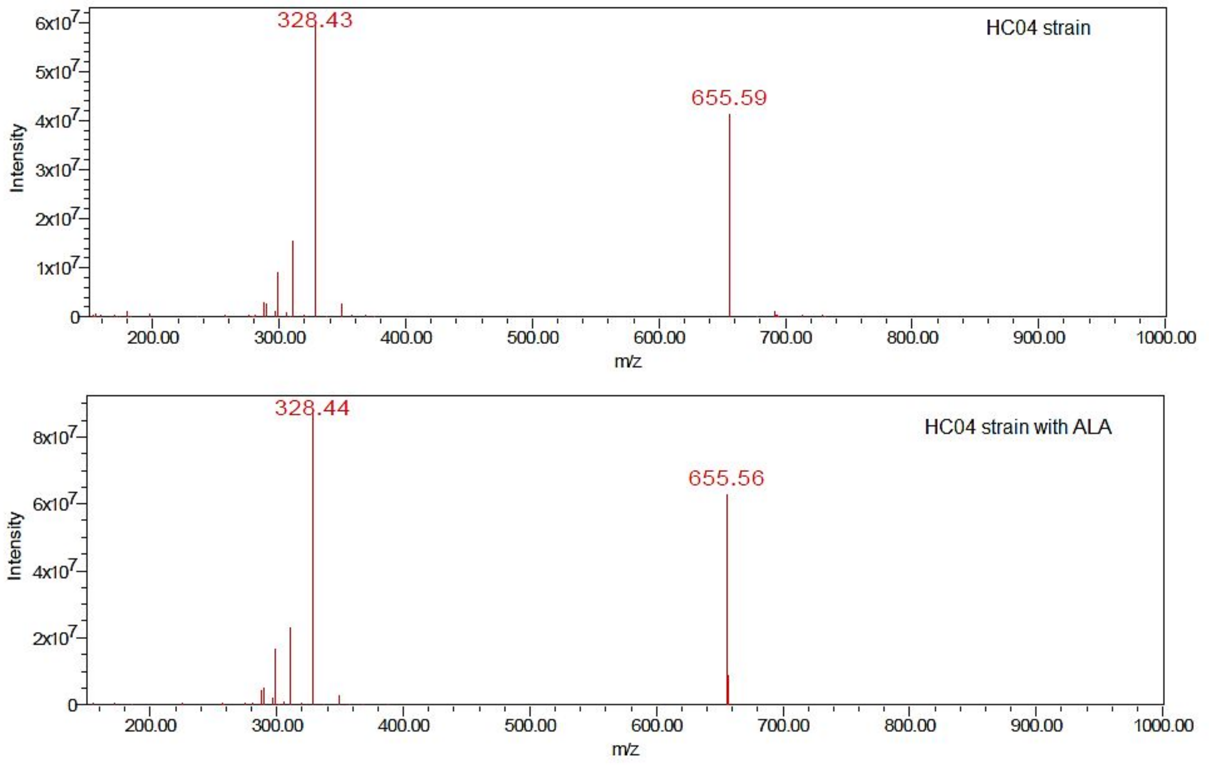

Figure S5. Enhanced product ion (+EPI) spectrum taken from TIC of porphyrin extracts from HC04 *C. diphtheriae* strain cultivated with and without ALA (2 mmol/L) addition. The spectrum shows the m/z precursor ion ($[M+H]^+$ = 655) and the doubly charged parent ion ($[M+2H]^{2+}$ = 328.5), both for coproporphyrin III.

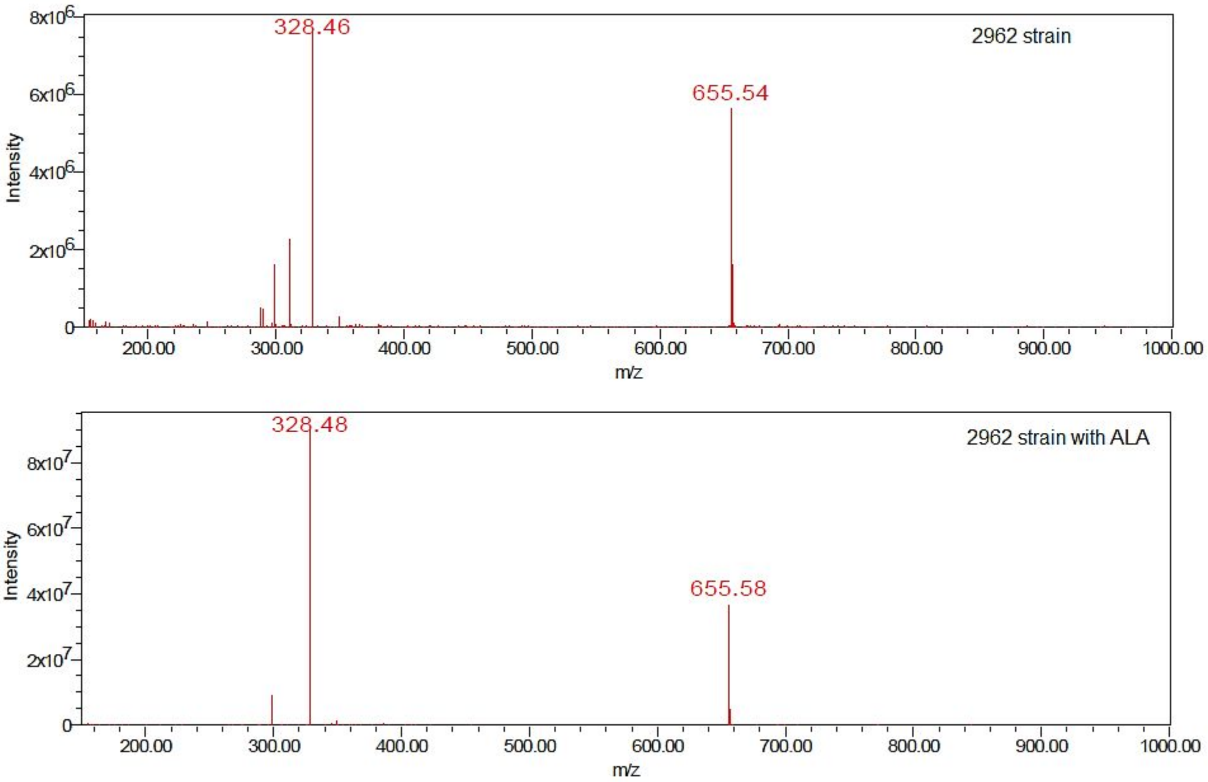

Figure S6. Enhanced product ion (+EPI) spectrum taken from TIC of porphyrin extracts from 2962 *C. diphtheriae* strain cultivated with and without ALA (2 mmol/L) addition. The spectrum shows the m/z precursor ion ($[M+H]^+$ = 655) and the doubly charged parent ion ($[M+2H]^{2+}$ = 328.5), both for coproporphyrin III.

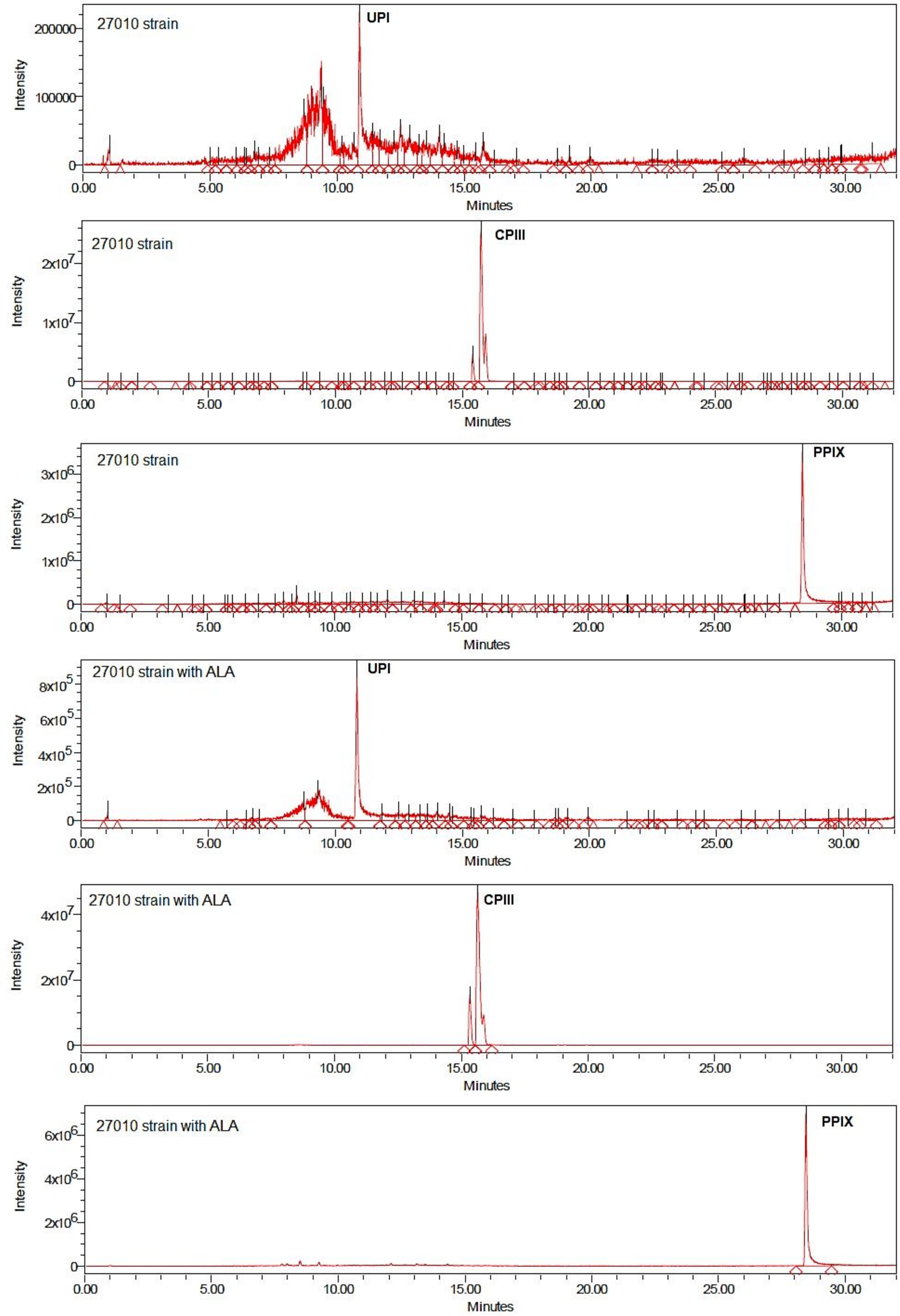

Figure S7. HPLC-MS chromatograms of the porphyrin extract from *C. diphtheriae* 27010 strain cultivated with and without ALA addition (2 mM). Chromatogram UPI is representative of uroporphyrin I (ion $[M+H]^+ = 830$); chromatogram CPIII is representative of coproporphyrin III (ion $[M+H]^+= 655$) and the chromatogram PPIX is representative of protoporphyrin ion $[M+H]^+= 563$).

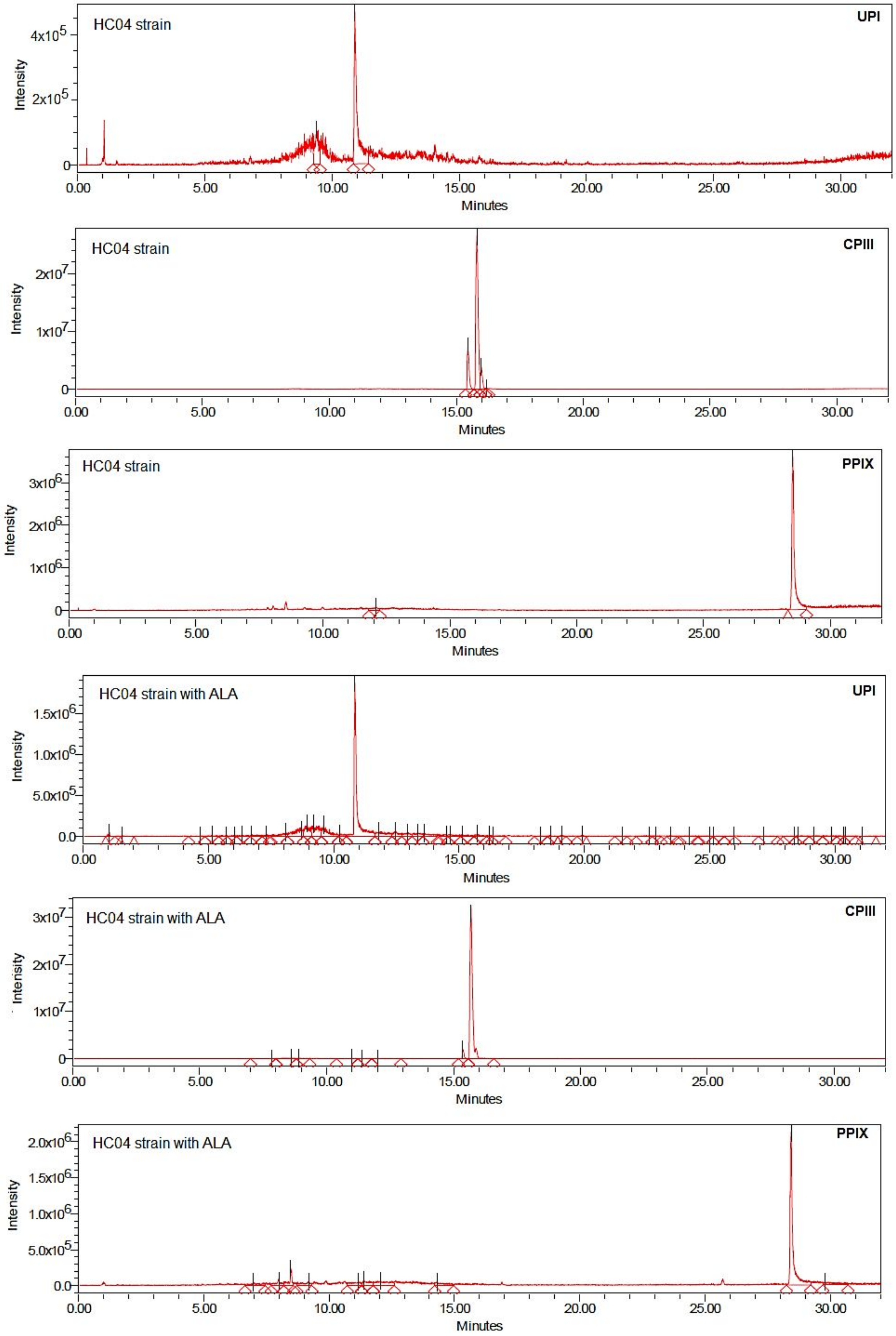

Figure S8. HPLC-MS chromatograms of the porphyrin extract from *C. diphtheriae* HC04 strain cultivated with and without ALA addition (2 mM). Chromatogram UPI is representative of uroporphyrin I (ion $[M+H]^+$ = 830); chromatogram CPIII is representative of coproporphyrin III (ion $[M+H]^+$= 655) and the chromatogram PPIX is representative of protoporphyrin ion $[M+H]^+$= 563

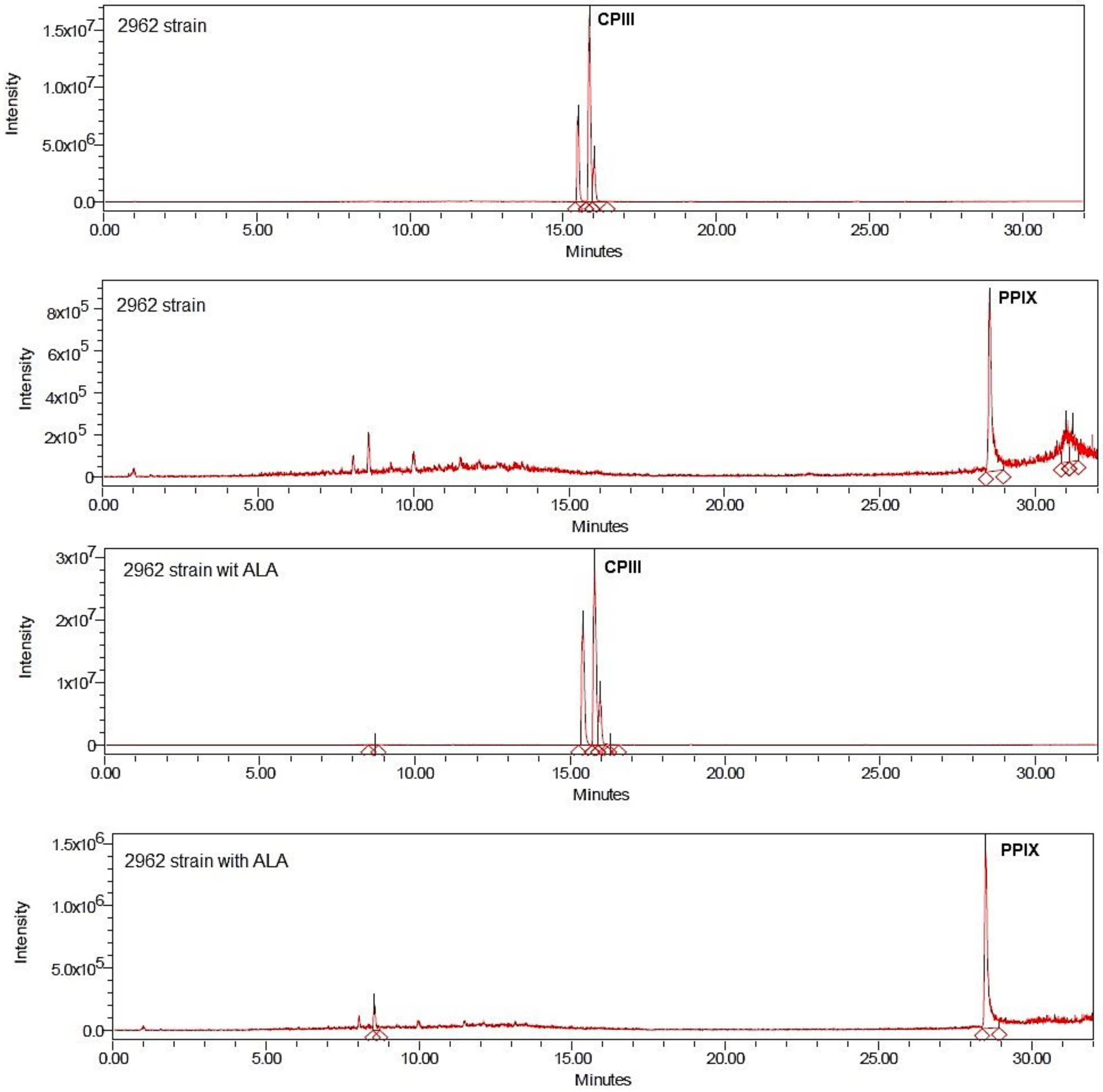

Figure S9. HPLC-MS chromatograms of the porphyrin extract from *C. diphtheriae* 2962 strain cultivated with and without ALA addition (2 mM). Chromatogram CPIII is representative of coproporphyrin III (ion $[M+H]^+$= 655) and the chromatogram PPIX is representative of protoporphyrin ion $[M+H]^+$= 563
